# Butin Attenuates Arthritis in Complete Freund’s Adjuvant-Treated Arthritic Rats: Possibly Mediated by Its Antioxidant and Anti-Inflammatory Actions

**DOI:** 10.3389/fphar.2022.810052

**Published:** 2022-02-15

**Authors:** Sami I. Alzarea, Abdullah F. Alasmari, Abdullah S. Alanazi, Abdulaziz I. Alzarea, Metab Alharbi, Abdulrahman Alshammari, Imran Kazmi, Fakhria A. Aljoufi, Nadeem Sayyed, Muhammad Afzal

**Affiliations:** ^1^ Department of Pharmacology, College of Pharmacy, Jouf University, Sakaka, Saudi Arabia; ^2^ Department of Pharmacology and Toxicology, College of Pharmacy, King Saud University, Riyadh, Saudi Arabia; ^3^ Department of Clinical Pharmay, College of Pharmacy, Jouf University, Sakaka, Saudi Arabia; ^4^ Health Science Research Unit, Sakaka, Saudi Arabia; ^5^ Department of Biochemistry, Faculty of Science, King Abdulaziz University, Jeddah, Saudi Arabia; ^6^ Clinical Research Department, Meril Life Sciences Pvt. Ltd., Vapi, India

**Keywords:** acetylcholinesterase, butin, flavonoids, neuroprotective, arthritis

## Abstract

The present research work was planned to evaluate the antioxidant and anti-inflammatory actions of butin in preventing complete Freund’s adjuvant-induced arthritis in rats. Adult Wistar rats (200–240 g) were segregated equally into four groups: Group I (normal) and Group II complete Freund’s adjuvant (CFA control) were administered orally with 3 ml/kg of 0.5% SCMC (vehicle); Group III and Group IV were test groups and orally administered 25 and 50 mg/kg of butin. These oral treatments were administered for a total of 21 days. In the 21-day treatment schedule, on the first day, animals from group I (normal control) were injected a single dose of normal saline (0.1 ml) intradermally into one of the hind paws, and animals from Group II to IV were injected CFA (0.1 ml) intradermally into one of the hind paws. During the treatment schedule, the volume of the hind paw and body weight were recorded at every 7 days intervals, and animals were scored for severe arthritis on days 17, 19, and 21. On the 22nd day, samples of blood were withdrawn by puncturing the retro-orbital sinus for analysis of RBC, WBC, hemoglobin, ALT, AST, ALP, PGE2, and cytokines. After blood withdrawal, animals were euthanized; the paw was separated by cutting at the ankle joint and used for analysis of oxidative stress and antioxidant parameters, as well as for the histopathological study. Administration of butin to CFA-treated animals significantly attenuated the CFA-induced inflammatory response, oxidative stress, and reversed the histopathological alteration towards normal. According to the findings, butin has anti-inflammatory and anti-arthritic properties in rats with CFA-induced arthritis.

## Introduction

Inflammation of joints and the synovial membrane is the characteristic feature of rheumatoid arthritis (RA), which can result in bone erosion and loss of joints (Alavala et al., 2020; Shams et al., 2021). Patients with this condition have symmetrical joint pain, morning stiffness, joint inflammation, cartilage and bone degeneration, and rheumatoid nodules under the skin (Chang et al., 2015; Basile et al., 2021). However, RA is linked to secondary amyloidosis, lymphomas, vasculitis, cardiovascular, pulmonary, psychiatric, and skeletal diseases, all of which can lead to permanent impairment in some cases (Basile et al., 2021; Shams et al., 2021). Smoking, gender (females are more likely), obesity, advanced age, and genetic makeup are risk factors for RA (Shams et al., 2021). This condition affects roughly 0.75% of the adult population in India and 1% worldwide, posing a severe health burden (Alavala et al., 2020; Shams et al., 2021).

Increased transcription factors and cytokine expressions are part of the mechanism that causes joint degeneration in RA (Zhu et al., 2016). Interleukins and tumor necrosis factor (TNF)-α have been linked to arthritis etiology (Zhu et al., 2016; Alavala et al., 2020). Inflammation is aided by IL-6, which stimulates blood vessel development. IL-1 promotes bone resorption and cartilage degradation by altering nitric oxide (NO) and prostaglandin (PGE2) production. PGE2 can create a fever by activating pain receptors (Campbell et al., 1990; Alavala et al., 2020). As a result of the imbalance between proinflammatory and anti-inflammatory states, synovial membrane inflammation and joint injury occurred (Chimenti et al., 2015; Alavala et al., 2020).

In the therapy of RA, steroid hormones, biological agents, immunosuppressants, and anti-inflammatory medications are employed (Lima-Garcia et al., 2011; Atzeni et al., 2013; Pincus and Cutolo, 2015; Alavala et al., 2020).

Anti-arthritic medications have a variety of significant benefits; however, their clinical use is limited due to reasons including high costs, adverse effects, and potency at a specific target region (Ekambaram et al., 2010; Alavala et al., 2020). Hormonal irregularities, decreased immunity, gastrointestinal tract abnormalities, and CVS difficulties have all been reported as side effects of RA (Petchi et al., 2015; Alavala et al., 2020). As a result, the therapeutic approach for arthritis necessitates a cost-effective medicine, has a long shelf life, and has few or no adverse effects.

In preclinical RA, various animal models are used; however, complete Freund’s adjuvant (CFA)-generated arthritis has some parallels to human patients, making it the ideal model for inducing arthritis (Ghosh et al., 2010; Alavala et al., 2020).

Many flavonoids such as theaflavin (Datta et al., 2014), pentahydroxy (Tang et al., 2021), Detralex (Rovenský et al., 2009), and total flavonoids of Astragalus (Liu et al., 2017) have been shown beneficial effects in adjuvant-induced arthritic rodents.

**FIGURE 1:**
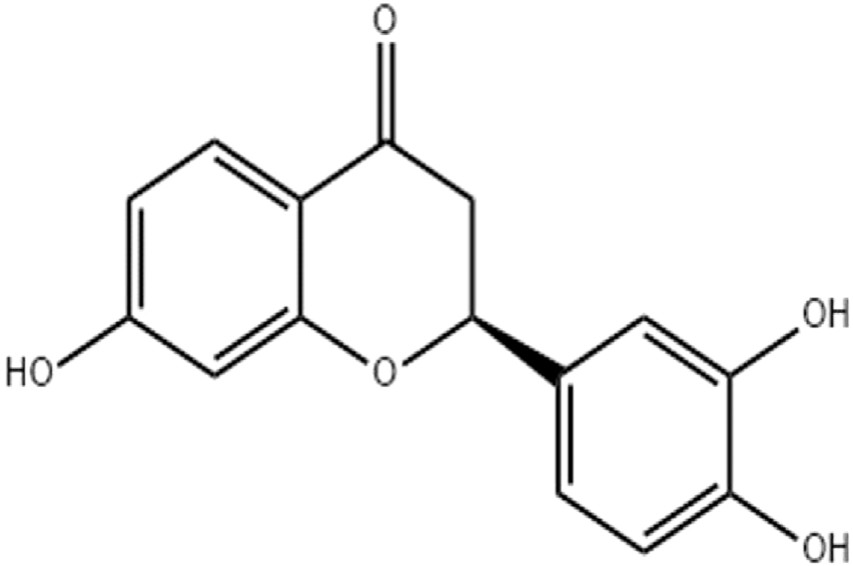
Butin (7,3′,4′-trihydroxydihydroflavone).

Butin (7,3′,4′-trihydroxydihydroflavone, Figure 1), a physiologically active flavonoid derived from plants such as *D. odorifera*, *R. verniciflua*, *V. anthelmintica*, and *A. mearnsii*, exhibits potent antioxidant, anti-inflammatory, and antiplatelet properties (Bhargava, 1986; Zhang et al., 2010; Zhang et al., 2011; Duan et al., 2017). By inhibiting the inflammatory pathway, butin (25 and 50 mg/kg) reduces brain edema in a rat model of intracerebral bleeding (Li and Jiwu, 2018). Based on the above facts, the current investigation was planned to evaluate the antioxidant and anti-inflammatory actions of butin in preventing complete Freund’s adjuvant (CFA)-induced arthritis in rats (Nagakannan et al., 2012).

## Materials and Methods

### Chemicals

CFA (Sigma-Aldrich, United States), 5, 50-dithiobis-2-nitrobenzoic acid, trichloroacetic acid, reduced glutathione, thiobarbituric acid (Hi-Media Laboratories Pvt., Ltd.), ALP, ALT, AST, IL-1β, IL-6, TNF-α, and the PGE2 estimation kit (Modern Lab, India) were used in the study.

### Animals

Adult Wistar rats (200–240 g) were acclimatized in the laboratory. They were free to access water and food. The Institutional Animal Ethics Committee authorized the experimental protocol and was following the guidelines of CPCSEA, Govt. of India (Binawade and Jagtap, 2013; C et al., 2014; Dhadde et al., 2016; Mehan et al., 2018).

### Experimental Design

Butin was diluted with 0.5% sodium CMC solution and given to experimental animals orally for 21 days. They were intradermally injected a single dosage of CFA (0.1 ml) into one of the hind paws of rats to develop arthritis (Alavala et al., 2020).

Adult Wistar rats (200–240 g) were equally (*n* = 6) segregated into four groups and given the following treatments: Groups I and II, normal and CFA control groups, received 0.5% sodium CMC (3 ml/kg/day) treatment for 21 days. The test groups III and IV were given butin (25 and 50 mg/kg, respectively) for 21 days. In the 21-day schedule, on the first day, normal saline (0.1 ml) was injected to group I animals (normal control) intradermally into the hind paw (right), and animals from Group II to IV were injected with a single dosage of CFA (100 µl) intradermally into the hind paw (Alavala et al., 2020). During the treatment schedule, the volume of the paw and body mass were recorded at every 7 days interval, and animals were scored for severe arthritis on days 17, 19, and 21.

On the 22nd day, samples of blood were withdrawn by puncturing the retro-orbital sinus for analysis of hematological and serum biochemical estimation. After blood withdrawal, animals were euthanized; the paw was isolated by cutting at the ankle joint and used for histopathological and biochemical assessments.

### Measurements of the Volume of the Paw and Body Weight

On day 1, the volume of the hind paw and body weight were measured before CFA treatment and subsequently at an interval of 7 days for next 21 days. The digital plethysmometer was used to measure the hind paw volume.

### Arthritis Severity Scoring

On a scale of 0 to 4, arthritis severity was analyzed and graded. Arthritis severity was graded in such a way that the parameters included the following: no swelling (grade 0), erythema in a finger or mild swelling (grade 1), swelling in fingers (grade 2), swelling of the ankle or wrist (grade 3), and significant arthritic swelling in wrist and fingers (grade 4).

### Estimation of the Blood Parameter

On day 22, samples of blood were collected into two separate tubes, one of which contained dipotassium EDTA as the anticoagulant. The blood was withdrawn by retro-orbital plexus puncture. The blood sample collected in a tube containing the anticoagulant was used for estimation of hemoglobin, RBC, and WBC, using a blood cell counter. The anticoagulant-free blood sample was centrifuged (2,500 × g, 10 min), and the serum was used for estimation of PGE2, aspartate aminotransferase (AST), alanine aminotransferase (ALT), alkaline phosphatase (ALP), and cytokines.

### Estimation of ALP, ALT, and AST

The contents of ALP, ALT, and AST in the serum were estimated using a biochemical diagnostic kit, as per kit manufacturer’s instructions, using a semi autoanalyzer. The contents of ALT, AST, and ALP were expressed in U/L of the serum.

### Estimation of PGE2

PGE2 levels were estimated using an ELISA kit by the competitive method. In a 96-well microtiter plate, 0.15 ml of samples, control, and standard were placed in precoated wells. After that, the PGE2 antibody (50 μl) was added and incubated at 37°C on a horizontal orbital microplate shaker (500 ± 50 rpm). After 60 min of incubation, the PGE2 conjugate (50 µl) was placed in each well and allowed incubation for next 2 h. After that, each well was filled with the substrate solution (200 μl) and incubated at room temperature for 30 min. Then, by adding the stop solution (100 μl), the reaction was halted, and at 450 nm, the optical density was measured.

### Estimation of Cytokines

The IL-1β, IL-6 and TNF-α (pro-inflammatory cytokines), and IL-10 (the anti-inflammatory cytokine) were estimated by ELISA kits, as per the kit. The amount of markers was calculated from standard curves and represented as pg/ml of the serum.

### Biochemical Estimations in Paw Tissue

#### Tissue Homogenization

The piece of subcutaneous tissue from the collected paw was cleaned using ice-cold isotonic saline and homogenized in ice-cold conditions in pH 7.4, 0.1 M phosphate buffer. The endogenous antioxidants and oxidative and nitrative stress markers were estimated in the supernatant of the homogenate.

#### Endogenous Antioxidants

The reduced glutathione (GSH) was quantified as per the Ellman method (Ellman, 1959). Using the Misra and Fridovich method, superoxide dismutase (SOD) was determined (Misra and Fridovich, 1972). To estimate the catalase activity, 100 µl of the sample was mixed with 1.9 ml of phosphate buffer (50 mM, pH 7.0) in the cuvette, and 1.0 ml of freshly prepared H_2_O_2_ (30 mM) was added as a reaction initiator (Aebi et al., 1974).

#### Oxidative and Nitrative Stress Markers

Malondialdehyde (MDA) was estimated in the tissue homogenate using the Wills method. The MDA level was represented as nmol/mg protein (Wills, 1966). The MPO levels were estimated as per the published method (Nagarjun et al., 2017). Griess reagent was used to estimate nitrite. The standard curve of sodium nitrite was used to calculate the nitrite content and expressed in µM/g tissue (Green et al., 1982; Slaoui et al., 2017).

#### Observation of Histopathological Changes

The isolated paw (skin, subcutaneous tissue along with the bone) samples (*n* = 3) were preserved in formalin (10%), cut into 5 mm thick sections by embedding in paraffin wax. For histological examinations, the slices were stained with the dye (hematoxylin and eosin) (Nagarjun et al., 2017; Slaoui et al., 2017). The stained tissues were analyzed for inflammatory alterations such as necrotic foci, cell infiltration, tissue structure destruction such as patches, damage to the nucleus, and so on.

### Statistics

Statistics was conducted by GraphPad Prism. The data are shown as a standard error of mean (S.E.M.). One-way ANOVA followed by Tukey’s test was also carried out. Scoring data were analyzed statistically using the Kruskal–Wallis non-parametric test. The significance threshold was set at *p* < 0.05.

## Results

### Body weight

**FIGURE 2:**
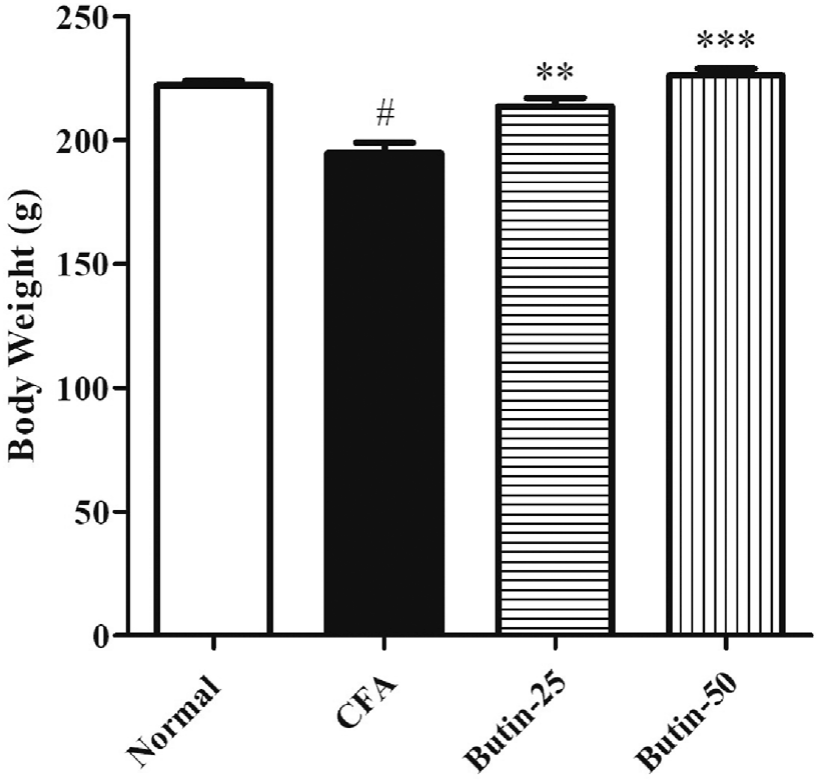
Effect of butin on body weight in CFA-treated rats. Values are expressed in mean ± SEM (*n* = 6). #*p* < 0.05 vs. normal control rats and **p < 0.01 and ***p < 0.001 vs CFA control rats. One-way ANOVA followed by Tukey’s *post hoc* test.

At the time of grouping, all the groups’ animal body weight was in the same range. Administration of CFA reduced 12.61% of the bodyweight in animals when compared to the normal control animals, and the values were statistically significant (*p* < 0.05). As butin was given to CFA-treated rats, the body weight increased by 9.75–16.7% than that of the CFA-control group, and the values were significant (*p* < 0.01 and *p* < 0.001). Figure 2 depicts the detailed body weight results.

### Paw Volume

**FIGURE 3:**
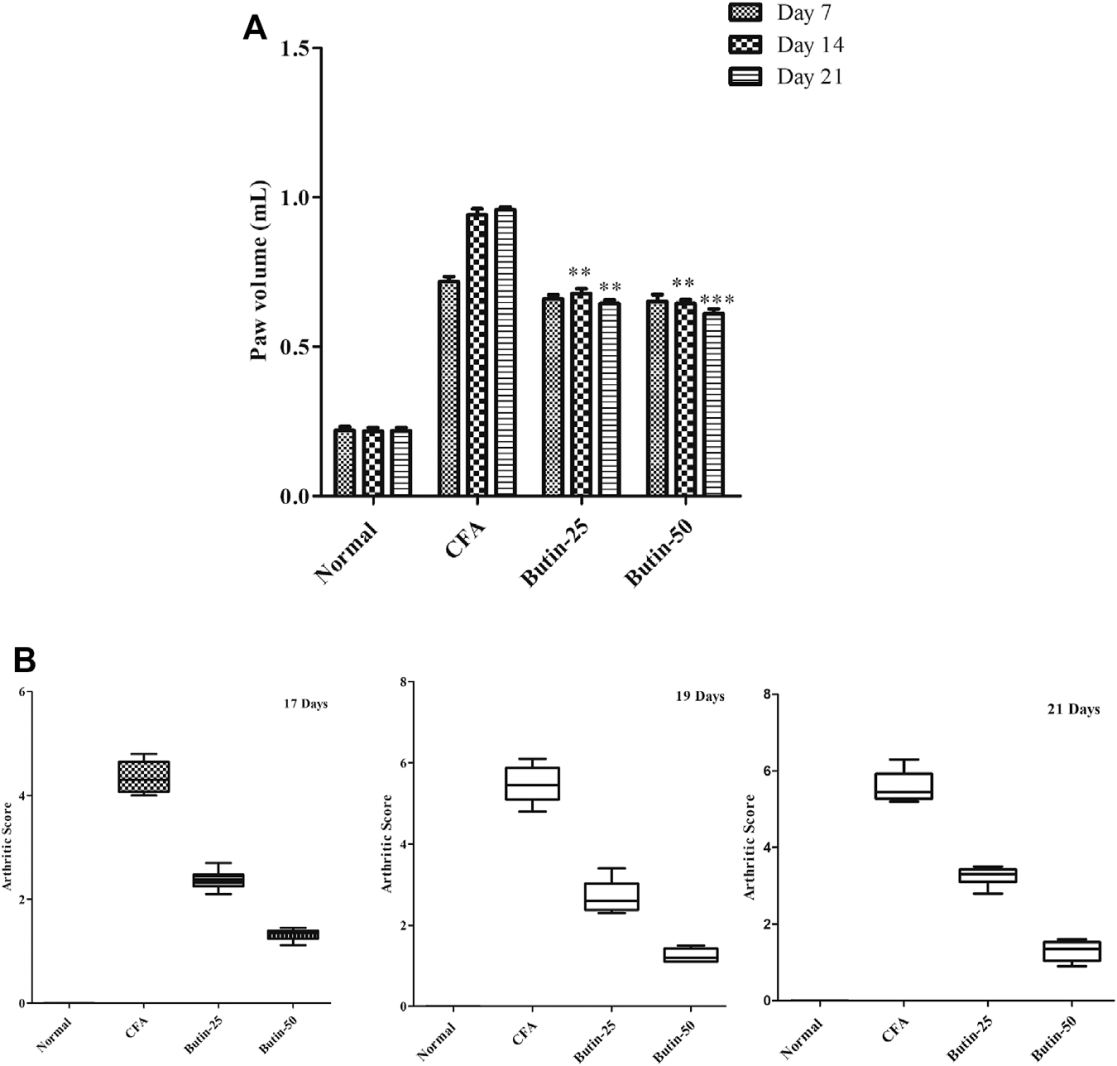
Effect of butin on **(A)** the paw volume and **(B)** arthritis severity score in CFA-treated rats. Values are expressed in mean ± SEM (*n* = 6). #*p* < 0.05 vs. normal control rats and **p < 0.01 and ***p < 0.001 vs CFA control rats. Two-way ANOVA followed by the Bonferroni *post hoc* test for paw volume and Kruskal–Wallis test for the arthritis severity score.

CFA produced edema in the paw and it was evidenced by 2–3-fold increased (*p* < 0.001) paw volume in all of the evaluated days in CFA-treated animals. Administration of butin (25 and 50 mg/kg) to CFA-treated animals attenuated the paw volume in all the tested intervals. The results were statistically significant *p* < 0.01 (on day 14) and *p* < 0.01 and *p* < 0.001 (on day 21). The results of the paw volumes are shown in Figure 3A.

### Arthritis Severity Score

When CFA-treated rats were compared to normal control rats, arthritis severity was dramatically elevated. The scores were statistically significant on all the tested days (*p* < 0.001). Administration of butin to the CFA-treated rats improved the condition of arthritis; hence, the animals get lower scores in the CFA-treated group than the CFA control group. The score values were significant on the 17th day, 19th day, and 21st day (*p* < 0.05) vs. CFA control group. The arthritis severity score results are shown in Figure 3B.

### Blood Parameter

#### Hematological Parameters

**FIGURE 4:**
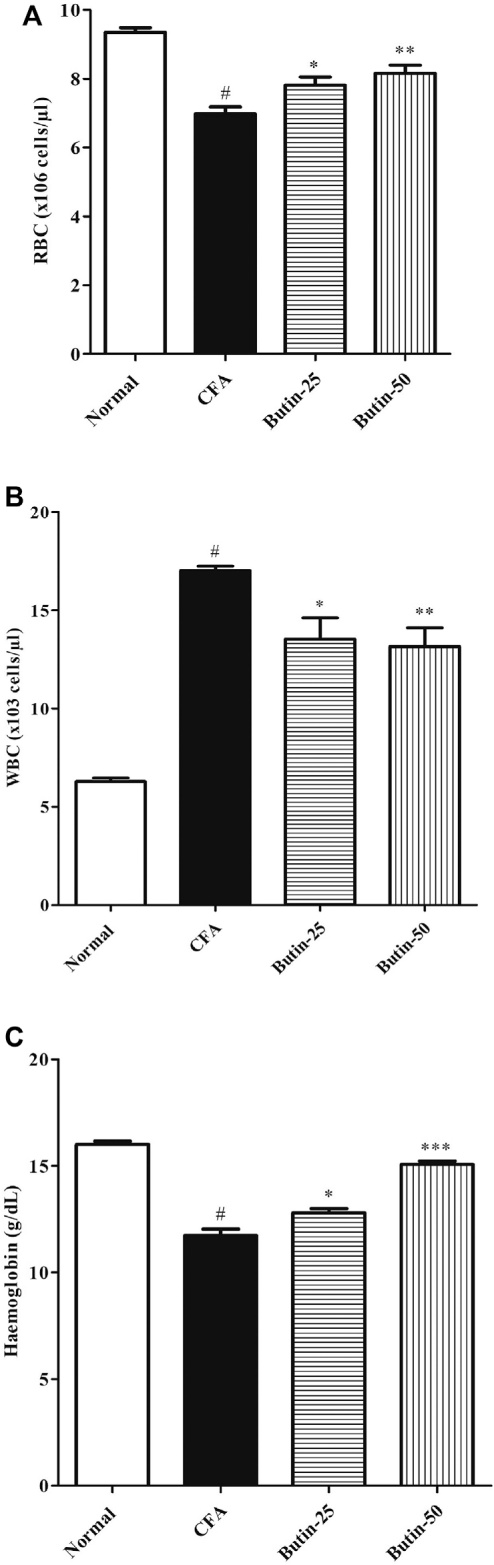
Effect of butin on **(A)** RBC count, **(B)** WBC count, and **(C)** Hemoglobin levels in CFA-treated rats. Values are expressed in mean ± SEM (*n* = 6). ^#^ *p* < 0.05 vs. normal control rats and ^**^ *p* < 0.01 and ^***^ *p* < 0.001 vs CFA control rats. One-way ANOVA followed by Tukey’s *post hoc* test.

Administration of CFA decreased 26.77% of hemoglobin levels (*p* < 0.05) and 25.34% of RBC counts (*p* < 0.05) and increased 170.88% of the WBC count (*p* < 0.05) in comparison with the normal control group, whereas butin attenuated the hematological changes induced by CFA; 9–28% of hemoglobin levels (*p* < 0.05 and *p* < 0.001) and 12–17% of RBC counts (*p* < 0.05 and *p* < 0.001) were increased, and 20% of WBC counts (*p* < 0.05 and *p* < 0.001) were decreased in CFA-treated rats. The results of hematological parameters are represented in Figure 4.

#### ALT, AST, and ALP

ALP, ALT, and AST levels in CFA-treated animals were substantially high (*p* < 0.05) vs. normal control animals. Butin (25 and 50 mg/kg) decreased the levels of ALT by 16.03–20.95% (*p* < 0.05 and *p* < 0.01), AST by 6.77–22.55% (*p* < 0.01 and *p* < 0.001), and ALP by 11.5–22.96% (*p* < 0.01 and *p* < 0.001) in CFA-treated rats vs. CFA control animals. Table 1 represented the ALT, AST, and ALP results.

**TABLE 1:** Effect of butin on liver enzymes (ALT, AST, and ALP) in experimental animals.

Groups	ALT (U/L)	AST (U/L)	ALP (U/L)
I (Normal control)	58.83 ± 1.52	80.66 ± 1.17	151 ± 1.47
II (CFA control)	91.5 ± 2.03	254 ± 0.74	310.16 ± 0.21
III (Butin 25 mg/kg)	76.83 ± 0.87^*^	236.83 ± 1.39^**^	274.5 ± 1.68^**^
IV (Butin 50 mg/kg)	72.33 ± 1.55^**^	196.66 ± 0.45^***^	239 ± 2.05^***^

ALT, alanine transaminase; AST, aspartate aminotransferase; ALP, alkaline phosphatase.

^#^ *p* < 0.05 vs. normal control rats and **p < 0.01 and ***p < 0.001 vs. CFA, control rats. One-way ANOVA followed by Tukey’s *post hoc* test.

#### PGE2 Levels

**FIGURE 5:**
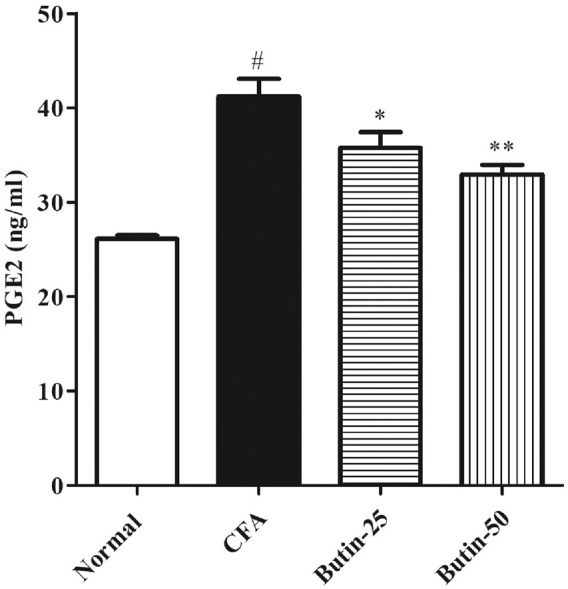
Effect of butin on PGE2 levels in CFA-treated rats. Values are expressed in mean ± SEM (*n* = 6). ^#^ *p* < 0.05 vs. normal control rats and ^**^ *p* < 0.01 and ^***^ *p* < 0.001 vs. CFA control rats. One-way ANOVA followed by Tukey’s *post hoc* test.

PGE2 levels in CFA-treated rats were higher (*p* < 0.05) vs. normal control rats. Butin (25 and 50 mg/kg) reduced the PGE2 levels by 13.18–20.2% in CFA-treated rats (*p* < 0.05 and *p* < 0.01) vs. CFA control animals. The PGE2 result is depicted in Figure 5.

#### Levels of Cytokines

**FIGURE 6:**
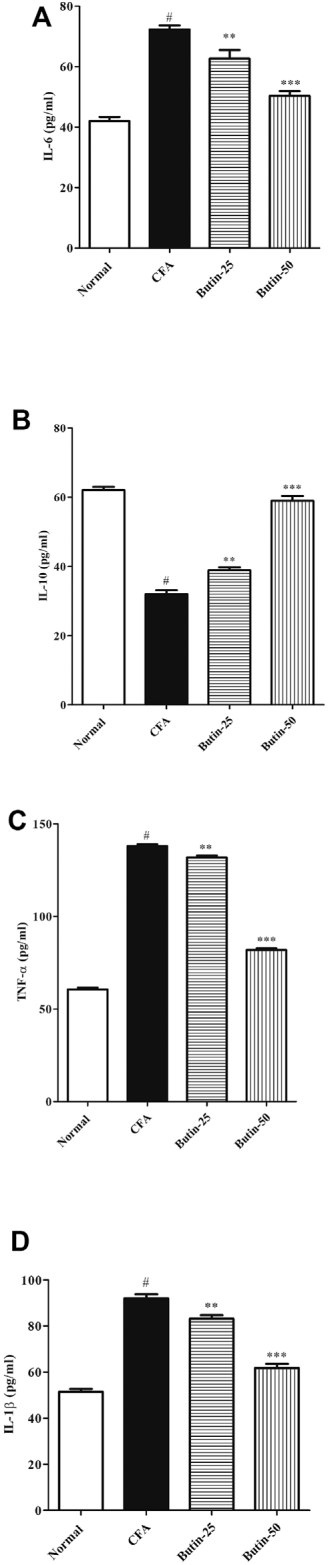
Effect of butin on **(A)** TNF-α, **(B)** IL-1β, **(C)** IL-6, and **(D)** IL-10 levels in CFA-treated rats. Values are expressed in mean ± SEM (*n* = 6). ^#^ *p* < 0.05 vs. normal control rats and ^**^ *p* < 0.01 and ^***^ *p* < 0.001 vs. CFA control rats. One-way ANOVA followed by Tukey’s *post hoc* test.

Proinflammatory cytokines IL-1β, IL-6, and TNF-α were considerably (*p* < 0.05) increased, and the anti-inflammatory cytokine IL-10 was significantly (*p* < 0.05) lowered in CFA-treated rats vs. normal control. Butin attenuated IL-1β (9.59–32.79%), IL-6 (13.35–30.41%), and TNF-α (4.49–40.70%) in CFA-treated rats with the significance value of *p* < 0.01 and *p* < 0.001 and improved IL-10 (21.34–84.37%) levels (*p* < 0.01 and *p* < 0.001) vs. the CFA control group. The results of cytokine estimations are shown in Figure 6.

### Biochemical Estimations in Paw Tissue

#### Endogenous Antioxidants

Administration of CFA disturbed the levels of endogenous antioxidants (catalase, GSH, and SOD). CFA control animals had lower levels (*p* < 0.05) of catalase, GSH, and SOD than normal control animals. Treatment with butin to CFA-injected animals restored GSH (41.49–58.53%), SOD (15–19.83%), and catalase (19–59.69%) levels (*p* < 0.05 and *p* < 0.01) toward normal. The endogenous antioxidant status results are shown in Table 2.

**TABLE 2:** Effect of butin on endogenous antioxidant levels (GSH, CAT, and SOD) in experimental rats.

Groups	GSH (mg/g of tissue)	CAT (nmol/min/ml)	SOD (U/ml)
I (Normal control)	1.39 ± 1.58	12.95 ± 2.11	12.56 ± 1.02
II (CFA control)	0.61 ± 0.75^#^	7.12 ± 0.97^#^	7.20 ± 0.89^#^
III (Butin 25 mg/kg)	0.87 ± 0.44^*^	8.47 ± 1.62^**^	8.18 ± 1.21^*^
IV (Butin 50 mg/kg)	0.97 ± 1.80^**^	11.37 ± 0.35^***^	8.61 ± 0.60^**^

GSH, glutathione; CAT catalase; SOD, superoxide dismutase.

#*p* < 0.05 vs normal control rats and ^**^ *p* < 0.01 and ^***^ *p* < 0.001 vs CFA, control rats. One-way ANOVA followed by Tukey’s *post hoc* test.

#### Markers of Nitrative and Oxidative Stress

**FIGURE 7:**
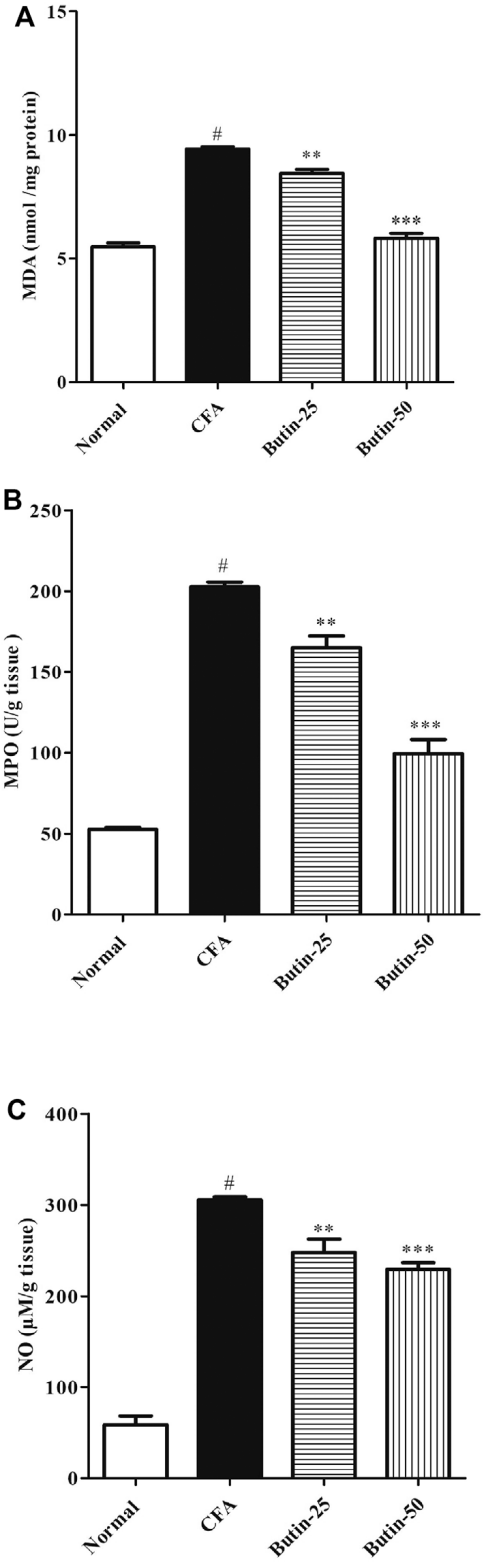
Effect of butin on oxidative and nitrative stress marker levels **(A)** MDA, **(B)** MPO, and **(C)** Nitrate in CFA-treated rats. Values are expressed in mean ± SEM (*n* = 6). ^#^ *p* < 0.05 vs. normal control rats and ^**^ *p* < 0.01 and ^***^ *p* < 0.001 vs. CFA control rats. One-way ANOVA followed by Tukey’s *post hoc* test.

CFA treatment increased (*p* < 0.05) the levels of MDA by 72.48%, MPO by four-folds, and nitrite by six-folds when compared with normal control animals. Butin therapy reduced the elevated levels of MDA by 10.4–38.26% (*p* < 0.01 and *p* < 0.001), MPO by 18.59–50.91% (*p* < 0.01 and *p* < 0.001), and nitrite by 18.88–24.97% (*p* < 0.01 and *p* < 0.001) vs. the CFA control group. The results of markers of nitrative and oxidative stress are shown in Figure 7.

### Histopathological Observations

**FIGURE 8:**
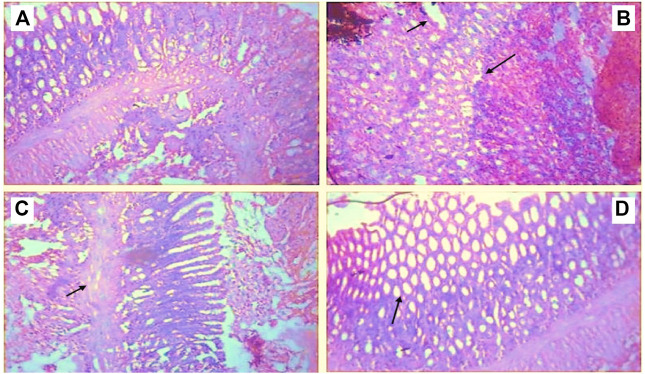
Histopathological observations of paw tissue in control and experimental groups **(A)** Normal control showed a normal structure of the paw tissue, **(B)** CFA control displayed inflamed cells with edema formation, **(C)** Butin 25 mg/kg, and **(D)** Butin 50 mg/kg. Both treatment groups showed almost a normal tissue structure with decreased inflammation and cell infiltration.

The hind paws of normal control animals have a consistent histological pattern. CFA treatment induced abnormalities in the hind paw such as edema, accumulation of inflammatory cells of the bone marrow, and cartilage destruction. Butin treatment at both doses reduced inflammatory symptoms, improved bone marrow, eliminated edema, and reduced cellular infiltration. The photographs of H&E staining of hind paw sections are shown in Figure 8.

## Discussion

Arthritis is linked with the loss of lean body mass and weight and that is known as rheumatoid cachexia (Patil et al., 2011). The large alterations in body mass seen in CFA-treated rats are consistent with previous research findings (Choudhary et al., 2014; Cui et al., 2019).

The arthritic score is a metric for measuring joint inflammation and is used to express the severity of arthritis (Choudhary et al., 2014). Butin-treated rats had significantly lower arthritic scores than FCA-treated rats, demonstrating that it has anti-inflammatory properties. Measuring paw swelling is a sensitive and quick way to figure out how much inflammation there is, as well as the therapeutic and curative effects of drugs (Kshirsagar et al., 2014). Administration of CFA elevated the paw volume of the rats, and this was in agreement with earlier reported studies (Kshirsagar et al., 2014; Alavala et al., 2020). On the other hand, treatment with butin to CFA-treated rats attenuated the CFA-induced paw swelling. This observation indicates the anti-inflammatory effect of butin against CFA-induced inflammation in the paw.

Increases in granulocytes and monocytes are linked to changes in the paw volume (Kshirsagar et al., 2014). The present study data were in agreement with this statement; there was an increased paw volume, and WBC levels were found in CFA-treated rats. Butin attenuated the CFA-induced increase in the paw volume and WBC levels, representing its anti-inflammatory effects against CFA in rats.

The cytokines are released when macrophages are activated in chronic inflammation and have been related to immunological arthritis. IL-6 is overexpressed at inflammatory sites, and it can play a crucial role in chronic inflammation (Kshirsagar et al., 2014; Alavala et al., 2020). IL-1, IL-6, and TNF-α increase acute-phase protein synthesis (Kshirsagar et al., 2014). It also triggers specific cellular and humoral immunological responses, such as B-cell differentiation and T-cell activation, among others. (Jazayeri et al., 2010; Kshirsagar et al., 2014). In CFA-treated rats, a considerable rise in TNF-α, IL-6, and IL-1β (proinflammatory cytokines) and a decrease in IL-10 (the anti-inflammatory cytokine) were observed. Administration of butin to CFA-injected rats attenuated the altered levels of cytokines toward normal, indicating the anti-inflammatory effect of butin.

Prostaglandins, which relax arteriolar smooth muscle cells and increase blood flow to the region, significantly amplify exudates (Kshirsagar et al., 2014). Injection of CFA elevated the PGE2 levels, and this may be responsible for an increased volume of rat paw. Treatment with butin reduced the elevated levels of PGE2 in CFA-injected rats, indicating its anti-inflammatory response against CFA.

The function of the oxidative state in the initiation of proinflammatory milieu in RA patients has been studied. The literature suggests that oxidative stress has a role in the development of RA (Quiñonez-Flores et al., 2016; Phull et al., 2018; da Fonseca et al., 2019). Oxidative stress is characterized as a hazardous scenario in which the pool of oxidative molecules has a negative equilibrium, favoring the prevalence of pro-oxidants (da Fonseca et al., 2019). MDA, MPO, and nitrite levels were all higher in the CFA-treated rats, showing that CFA caused oxidative stress. Treatment with butin in CFA-treated rats attenuated the levels of MDA, MPO, and nitrite in paw tissue (skin with subcutaneous tissue), indicating its inhibitory actions against CFA-induced oxidative stress in the rats.

Antioxidants can scavenge ROS/RNS and inhibit the oxidation process in cells; they act as regulatory players (da Fonseca et al., 2019; Fukai and Ushio-Fukai, 2011). Treatment with butin restored the endogenous antioxidants in CFA-treated rats in comparison with CFA control rats, indicating its antioxidant role against the CFA-induced oxidative insults.

The results of histopathological observations well support the anti-inflammatory and antioxidant properties of butin against CFA-induced inflammation and arthritis. Treatment with butin to CFA-treated rats reversed the CFA-induced inflammatory response and tissue architecture toward normal. Butin appears to have anti-inflammatory and anti-arthritic properties in rats when exposed to CFA.

## Conclusion

The results of this study show that butin has anti-inflammatory and anti-arthritic properties in rats with CFA-induced arthritis. It was demonstrated by the attenuation of observational, biochemical, and histological data in CFA-treated rats. Butin’s anti-CFA benefits can be ascribed to its antioxidant and anti-inflammatory characteristics. In the future, more research is needed to confirm the exact mechanism and its application in therapeutic situations (Nagakannan et al., 2012).

## Data Availability

The raw data supporting the conclusions of this article will be made available by the authors, without undue reservation.

## References

[B1] Aebi H. Wyss S. R. Scherz B. Skvaril F. (1974). Heterogeneity of Erythrocyte Catalase II. Isolation and Characterization of normal and Variant Erythrocyte Catalase and Their Subunits. Eur. J. Biochem. 48 (1), 137–145. 10.1111/j.1432-1033.1974.tb03751.x 4141308

[B2] Alavala S. Nalban N. Sangaraju R. Kuncha M. Jerald M. K. Kilari E. K. (2020). Anti-inflammatory Effect of Stevioside Abates Freund's Complete Adjuvant (FCA)-induced Adjuvant Arthritis in Rats. Inflammopharmacology 28 (6), 1579–1597. 10.1007/s10787-020-00736-0 32617791

[B3] Atzeni F. Benucci M. Sallì S. Bongiovanni S. Boccassini L. Sarzi-Puttini P. (2013). Different Effects of Biological Drugs in Rheumatoid Arthritis. Autoimmun. Rev. 12 (5), 575–579. 10.1016/j.autrev.2012.10.020 23219774

[B4] Basile M. S. Ciurleo R. Bramanti A. Petralia M. C. Fagone P. Nicoletti F. (2021). Cognitive Decline in Rheumatoid Arthritis: Insight into the Molecular Pathogenetic Mechanisms. Int. J. Mol. Sci. 22 (3), 1185. 10.3390/ijms22031185 33530359 PMC7865873

[B5] Bhargava S. K. (1986). Estrogenic and Postcoital Anticonceptive Activity in Rats of Butin Isolated from Butea Monosperma Seed. J. Ethnopharmacol 18 (1), 95–101. 10.1016/0378-8741(86)90046-2 3821138

[B6] Binawade Y. Jagtap A. (2013). Neuroprotective Effect of Lutein against 3-nitropropionic Acid-Induced Huntington's Disease-like Symptoms: Possible Behavioral, Biochemical, and Cellular Alterations. J. Med. Food 16 (10), 934–943. 10.1089/jmf.2012.2698 24138168

[B7] C J. H M M. Dhadde S. B. Durg S. Potadar P. P. B S T. (2014). Piroxicam Attenuates 3-nitropropionic Acid-Induced Brain Oxidative Stress and Behavioral Alteration in Mice. Toxicol. Mech. Methods 24 (9), 672–678. 10.3109/15376516.2014.961216 25191831

[B8] Campbell I. K. Piccoli D. S. Hamilton J. A. (1990). Stimulation of Human Chondrocyte Prostaglandin E2 Production by Recombinant Human Interleukin-1 and Tumour Necrosis Factor. Biochim. Biophys. Acta 1051 (3), 310–318. 10.1016/0167-4889(90)90140-9 2310781

[B9] Chang X. He H. Zhu L. Gao J. Wei T. Ma Z. (2015). Protective Effect of Apigenin on Freund's Complete Adjuvant-Induced Arthritis in Rats via Inhibiting P2X7/NF-Κb Pathway. Chem. Biol. Interact 236, 41–46. 10.1016/j.cbi.2015.04.021 25935278

[B10] Chimenti M. S. Triggianese P. Conigliaro P. Candi E. Melino G. Perricone R. (2015). The Interplay between Inflammation and Metabolism in Rheumatoid Arthritis. Cell Death Dis 6 (9), e1887. 10.1038/cddis.2015.246 26379192 PMC4650442

[B11] Choudhary M. Kumar V. Gupta P. Singh S. (2014). Investigation of Antiarthritic Potential of *Plumeria alba* L. Leaves in Acute and Chronic Models of Arthritis. Biomed. Res. Int. 2014, 474616. 10.1155/2014/474616 25025056 PMC4082847

[B12] Cui X. Wang R. Bian P. Wu Q. Seshadri V. D. D. Liu L. (2019). Evaluation of Antiarthritic Activity of Nimbolide against Freund's Adjuvant Induced Arthritis in Rats. Artif. Cell Nanomed Biotechnol 47 (1), 3391–3398. 10.1080/21691401.2019.1649269 31394949

[B13] da Fonseca L. J. S. Nunes-Souza V. Goulart M. O. F. Rabelo L. A. (2019). Oxidative Stress in Rheumatoid Arthritis: What the Future Might Hold Regarding Novel Biomarkers and Add-On Therapies. Oxid Med. Cel Longev 2019, 7536805. 10.1155/2019/7536805 PMC6942903 31934269

[B14] Datta P. Mukherjee S. Dasgupta S. C. Gomes A. Gomes A. (2014). Anti Arthritic Activity of Theaflavin (TF), Chief Flavonoid of Black tea against Adjuvant Induced Rheumatoid Arthritis in Experimental Animal Models. Orient Pharm. Exp. Med. 14 (3), 245–253. 10.1007/s13596-013-0144-0

[B15] Dhadde S. B. Nagakannan P. Roopesh M. Anand Kumar S. R. Thippeswamy B. S. Veerapur V. P. (2016). Effect of Embelin against 3-nitropropionic Acid-Induced Huntington's Disease in Rats. Biomed. Pharmacother. 77, 52–58. 10.1016/j.biopha.2015.11.009 26796265

[B16] Duan J. Guan Y. Mu F. Guo C. Zhang E. Yin Y. (2017). Protective Effect of Butin against Ischemia/reperfusion-Induced Myocardial Injury in Diabetic Mice: Involvement of the AMPK/GSK-3β/Nrf2 Signaling Pathway. Sci. Rep. 7, 41491. 10.1038/srep41491 28128361 PMC5269748

[B17] Ekambaram S. Perumal S. S. Subramanian V. (2010). Evaluation of Antiarthritic Activity of Strychnos Potatorum Linn Seeds in Freund's Adjuvant Induced Arthritic Rat Model. BMC Complement. Altern. Med. 10, 56. 10.1186/1472-6882-10-56 20939932 PMC2978115

[B18] Ellman G. L. (1959). Tissue Sulfhydryl Groups. Arch. Biochem. Biophys. 82 (1), 70–77. 10.1016/0003-9861(59)90090-6 13650640

[B19] Fukai T. Ushio-Fukai M. (2011). Superoxide Dismutases: Role in Redox Signaling, Vascular Function, and Diseases. Antioxid. Redox Signal. 15 (6), 1583–1606. 10.1089/ars.2011.3999 21473702 PMC3151424

[B20] Ghosh S. Mehla R. K. Sirohi S. K. Roy B. (2010). The Effect of Dietary Garlic Supplementation on Body Weight Gain, Feed Intake, Feed Conversion Efficiency, Faecal Score, Faecal Coliform Count and Feeding Cost in Crossbred Dairy Calves. Trop. Anim. Health Prod. 42 (5), 961–968. 10.1007/s11250-009-9514-5 20012194

[B21] Green L. C. Wagner D. A. Glogowski J. Skipper P. L. Wishnok J. S. Tannenbaum S. R. (1982). Analysis of Nitrate, Nitrite, and [15N]nitrate in Biological Fluids. Anal. Biochem. 126 (1), 131–138. 10.1016/0003-2697(82)90118-x 7181105

[B22] Jazayeri J. A. Carroll G. J. Vernallis A. B. (2010). Interleukin-6 Subfamily Cytokines and Rheumatoid Arthritis: Role of Antagonists. Int. Immunopharmacol 10 (1), 1–8. 10.1016/j.intimp.2009.09.019 19804846

[B23] Kshirsagar A. D. Panchal P. V. Harle U. N. Nanda R. K. Shaikh H. M. (2014). Anti-Inflammatory and Antiarthritic Activity of Anthraquinone Derivatives in Rodents. Int. J. Inflam 2014, 690596. 10.1155/2014/690596 25610704 PMC4290027

[B24] Li P. Jiwu C. (2018). Butin Attenuates Brain Edema in a Rat Model of Intracerebral Hemorrhage by Anti Inflammatory Pathway. Transl Neurosci. 9 (1), 7–12. 10.1515/tnsci-2018-0002 29755784 PMC5941697

[B25] Lima-Garcia J. F. Dutra R. C. da Silva K. Motta E. M. Campos M. M. Calixto J. B. (2011). The Precursor of Resolvin D Series and Aspirin-Triggered Resolvin D1 Display Anti-hyperalgesic Properties in Adjuvant-Induced Arthritis in Rats. Br. J. Pharmacol. 164 (2), 278–293. 10.1111/j.1476-5381.2011.01345.x 21418187 PMC3174409

[B26] Liu X. Y. Xu L. Wang Y. Li J. X. Zhang Y. Zhang C. (2017). Protective Effects of Total Flavonoids of Astragalus against Adjuvant-Induced Arthritis in Rats by Regulating OPG/RANKL/NF-κB Pathway. Int. Immunopharmacol 44, 105–114. 10.1016/j.intimp.2017.01.010 28092862

[B27] Mehan S. Monga V. Rani M. Dudi R. Ghimire K. (2018). Neuroprotective Effect of Solanesol against 3-nitropropionic Acid-Induced Huntington's Disease-like Behavioral, Biochemical, and Cellular Alterations: Restoration of Coenzyme-Q10-Mediated Mitochondrial Dysfunction. Indian J. Pharmacol. 50 (6), 309–319. 10.4103/ijp.IJP_11_18 30783323 PMC6364342

[B28] Misra H. P. Fridovich I. (1972). The Role of Superoxide Anion in the Autoxidation of Epinephrine and a Simple Assay for Superoxide Dismutase. J. Biol. Chem. 247 (10), 3170–3175. 10.1016/s0021-9258(19)45228-9 4623845

[B29] Nagakannan P. Shivasharan B. D. Thippeswamy B. S. Veerapur V. P. (2012). Restoration of Brain Antioxidant Status by Hydroalcoholic Extract of Mimusops Elengi Flowers in Rats Treated with Monosodium Glutamate. J. Environ. Pathol. Toxicol. Oncol. 31 (3), 213–221. 10.1615/jenvironpatholtoxicoloncol.v31.i3.30 23339696

[B30] Nagarjun S. Dhadde S. B. Veerapur V. P. Thippeswamy B. S. Chandakavathe B. N. (2017). Ameliorative Effect of Chromium-D-Phenylalanine Complex on Indomethacin-Induced Inflammatory Bowel Disease in Rats. Biomed. Pharmacother. 89, 1061–1066. 10.1016/j.biopha.2017.02.042 28292014

[B31] Patil C. R. Rambhade A. D. Jadhav R. B. Patil K. R. Dubey V. K. Sonara B. M. (2011). Modulation of Arthritis in Rats by Toxicodendron Pubescens and its Homeopathic Dilutions. Homeopathy 100 (3), 131–137. 10.1016/j.homp.2011.01.001 21784329

[B32] Petchi R. R. Parasuraman S. Vijaya C. Gopala Krishna S. V. Kumar M. K. (2015). Antiarthritic Activity of a Polyherbal Formulation against Freund's Complete Adjuvant Induced Arthritis in Female Wistar Rats. J. Basic Clin. Pharm. 6 (3), 77–83. 10.4103/0976-0105.160738 26229343 PMC4513335

[B33] Phull A. R. Nasir B. Haq I. U. Kim S. J. (2018). Oxidative Stress, Consequences and ROS Mediated Cellular Signaling in Rheumatoid Arthritis. Chem. Biol. Interact 281, 121–136. 10.1016/j.cbi.2017.12.024 29258867

[B34] Pincus T. Cutolo M. (2015). Clinical Trials Documenting the Efficacy of Low-Dose Glucocorticoids in Rheumatoid Arthritis. Neuroimmunomodulation 22 (1-2), 46–50. 10.1159/000362734 25227901

[B35] Quiñonez-Flores C. M. González-Chávez S. A. Del Río Nájera D. Pacheco-Tena C. (2016). Oxidative Stress Relevance in the Pathogenesis of the Rheumatoid Arthritis: A Systematic Review. Biomed. Res. Int. 2016, 6097417. 10.1155/2016/6097417 27340664 PMC4906181

[B36] Rovenský J. Stančíková M. Rovenská E. Štvrtina S. Štvrtinová V. Švík K. (2009). Treatment of Rat Adjuvant Arthritis with Flavonoid (Detralex), Methotrexate, and Their Combination. Ann. N Y Acad. Sci. 1173 (1), 798–804. 10.1111/j.1749-6632.2009.04618.x 19758231

[B37] Shams S. Martinez J. M. Dawson J. R. D. Flores J. Gabriel M. Garcia G. (2021). The Therapeutic Landscape of Rheumatoid Arthritis: Current State and Future Directions. Front. Pharmacol. 12, 680043. 10.3389/fphar.2021.680043 34122106 PMC8194305

[B38] Slaoui M. Bauchet A. L. Fiette L. (2017). Tissue Sampling and Processing for Histopathology Evaluation. Methods Mol. Biol. 1641, 101–114. 10.1007/978-1-4939-7172-5_4 28748459

[B39] Tang Y. Xie D. Gong W. Wu H. Qiang Y. (2021). Pentahydroxy Flavonoid Isolated from Madhuca Indica Ameliorated Adjuvant-Induced Arthritis via Modulation of Inflammatory Pathways. Sci. Rep. 11 (1), 17971. 10.1038/s41598-021-97474-2 34504248 PMC8429448

[B40] Wills E. D. (1966). Mechanisms of Lipid Peroxide Formation in Animal Tissues. Biochem. J. 99 (3), 667–676. 10.1042/bj0990667 5964963 PMC1265056

[B41] Zhang R. Kang K. A. Piao M. J. Chang W. Y. Maeng Y. H. Chae S. (2010). Butin Reduces Oxidative Stress-Induced Mitochondrial Dysfunction via Scavenging of Reactive Oxygen Species. Food Chem. Toxicol. 48 (3), 922–927. 10.1016/j.fct.2010.01.001 20060874

[B42] Zhang R. Lee I. K. Piao M. J. Kim K. C. Kim A. D. Kim H. S. (2011). Butin (7,3',4'-trihydroxydihydroflavone) Reduces Oxidative Stress-Induced Cell Death via Inhibition of the Mitochondria-dependent Apoptotic Pathway. Int. J. Mol. Sci. 12 (6), 3871–3887. 10.3390/ijms12063871 21747713 PMC3131597

[B43] Zhu L. Chen T. Chang X. Zhou R. Luo F. Liu J. (2016). Salidroside Ameliorates Arthritis-Induced Brain Cognition Deficits By regulating Rho/ROCK/NF-κB Pathway. Neuropharmacology 103, 134–142. 10.1016/j.neuropharm.2015.12.007 26690894

